# Liver Transplant Patients with High Preoperative Serum Bilirubin Levels Are at Increased Risk of Postoperative Delirium: A Retrospective Study

**DOI:** 10.3390/jcm9051591

**Published:** 2020-05-24

**Authors:** Kyu Hee Park, Hyo Jung Son, Yoon Ji Choi, Gene Hyun Park, Yoon Sook Lee, Ju Yeon Park, Hyun-Su Ri, Jae Ryong Shim

**Affiliations:** 1 Department of Pediatrics, Korea University Hospital, Ansan 15355, Korea; czrabbit@korea.ac.kr; 2 Department of Anesthesiology and Pain Medicine, National Police Hospital, Seoul 05715, Korea; gidget80@police.go.kr; 3 Department of Anaesthesiology and Pain Medicine, Ansan Hospital, Korea University College of Medicine, Ansan 425020, Korea; ilock94@gmail.com (G.H.P.); yslee4719@gmail.com (Y.S.L.); 4 Department of Anesthesiology and Pain Medicine, Daedong Hospital, Busan 47737, Korea; monojp@naver.com; 5 Department of Anaesthesia and Pain Medicine, Pusan National University Yangsan Hospital, Yangsan 50612, Korea; johnri@naver.com; 6 Division of Hepato-Biliary-Pancreatic Surgery and Transplantation, Department of Surgery, Pusan National University Yangsan Hospital, Pusan National University School of Medicine, Yangsan 50612, Korea; zombiepr@nate.com

**Keywords:** bilirubin, complication, liver transplantation, postoperative delirium

## Abstract

Postoperative delirium is a common complication after liver transplantation (LT). A high model for end-stage liver disease (MELD) score is an independent risk factor for postoperative delirium, but it is unclear which of the components of this score are risk indicators. The aim of this study was to analyze the incidence of postoperative delirium according to the preoperative serum bilirubin level, a component of the MELD score, in patients who underwent LT. The medical records of 325 patients who underwent LT from January 2010 to February 2019 at a single university hospital were retrospectively reviewed. The patients were divided into two groups: those who experienced postoperative delirium (Delirium group, *n* = 69) and those who did not (Control group, *n* = 256). Data on the patients’ demographic characteristics, perioperative management, and postoperative complications were collected. Mean preoperative bilirubin level was higher in the Delirium group than in the Control group (*p* < 0.0001). In the Delirium group, 54 (78.26%) patients had preoperative bilirubin levels above 3.5 mg/dL. In the multivariate analysis, preoperative bilirubin above 3.5 mg/dL was associated with postoperative delirium (*p* = 0.002). Therefore, preoperative hyperbilirubinemia is an independent risk factor for postoperative delirium.

## 1. Introduction

Postoperative delirium is a common complication after liver transplantation (LT), and the reported incidence of this condition varies from 9% to 32% [1,2,3]. Delirium after LT can be caused by prior hepatic encephalopathy (HE), metabolic impairments including serum sodium changes or hypomagnesemia, severe ascites, and immunosuppressants [2,4,5,6]. Postoperative delirium is associated with high post-transplant mortality and morbidity and increased medical expenses due to prolonged intensive care unit (ICU) and hospital stays [7,8]. Postoperative delirium results in cognitive impairment, which typically lasts several days to weeks, although the course of this condition is usually reversible. Therefore, it is important to identify patients at high risk of postoperative delirium.

Recently, it was reported that delirium occurs frequently in patients with a high model for end-stage liver disease (MELD) score [9]. Bilirubin, one of the factors that calculates the MELD score, has both neuroprotective and neurotoxic effects [10]. Neurological symptoms caused by hyperbilirubinemia are common in newborns and typically reduce/disappear upon maturation of the blood–brain barrier [11]. However, that can increase in the case of liver cirrhosis or other severe hepatic impairment patients with decreased albumin production, or drug-induced bilirubin displacement on the albumin binding sites. In addition, the anesthesia/surgery might induce an blood–brain barrier dysfunction [12]. Therefore, the purpose of our study was to investigate whether preoperative serum bilirubin level is associated with the incidence of delirium after LT.

## 2. Experimental Section

### 2.1. Patient Population

**Figure 1:**
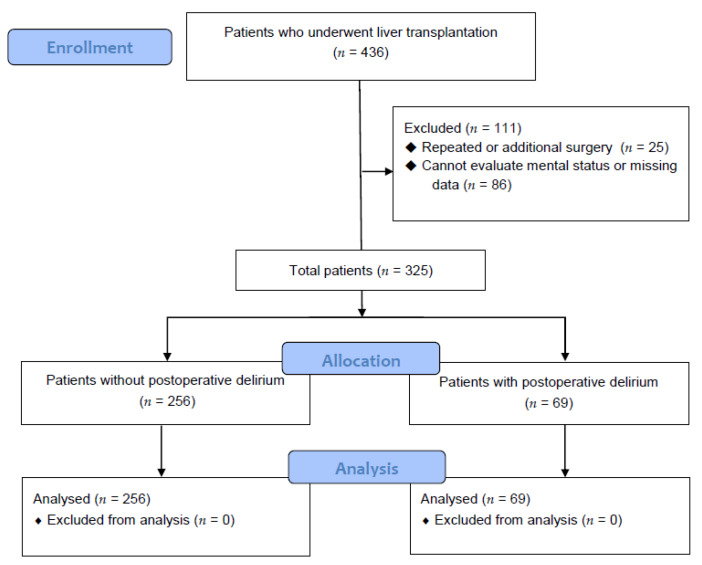
Flow diagram analyzing the effect of bilirubin on postoperative delirium in liver transplant patients.

This study was approved by the research committee of the hospital clinical research committee (5-2019-033) and the requirement for written informed consent was waived by the IRB. Computerized data were collected from the medical records of 325 patients who underwent LT from January 2010 to February 2019 (Figure 1). A total of 436 patients were initially recruited, but 111 were excluded because they underwent re-transplantation or additional surgery, did not have their mental status evaluated, or had data missing from their records. Many patients undergoing or receiving hepatic encephalopathy were not included in the study because there was no accurate record of consciousness levels prior to surgery. All patients treated for hepatic encephalopathy just before surgery were also excluded for the same reason.

### 2.2. Data Collection

In this study, patients were divided into two groups: those who experienced postoperative delirium (Delirium group, *n* = 69) and those who did not (Control group, *n* = 256). The patients’ basic demographic information and pre-, peri-, and postoperative laboratory and treatment data were collected. The patients’ existing records, preoperative evaluation records, anesthesia records, ICU records, progress notes, hospital nursing records, consult papers, and discharge records were reviewed retrospectively. Perioperative anesthetic management was also assessed. Preoperative laboratory tests were defined as tests performed in the 24 h prior to surgery. Postoperative laboratory tests were defined as tests performed in the first 24 h after surgery/performed on postoperative day 1. The infection rate was reviewed in cases with surgical site infection and sepsis.

### 2.3. Definition of Delirium

In the medical records, a patient was defined as having postoperative delirium if they had signs and symptoms consistent with the Diagnostic and Statistical Manual of Mental Disorders-IV criteria for delirium and were treated with haloperidol or other medication or if they were diagnosed by a psychiatrist using the Mini-Mental Status Examination and Confusion Assessment Method.

### 2.4. Intraoperative Protocol

General anesthesia was induced using 1–2 mg/kg of propofol and a muscle relaxant such as 0.6–1 mg/kg of rocuronium or 0.1–0.2 mg/kg of cisatracurium. Inhaled sevoflurane or desflurane was administered in an oxygen/gas mixture with 40–50% oxygen. Remifentanil (0.5–10 µg/kg/h) and a muscle relaxant (0.3–0.6 mg/kg/h of rocuronium or 0.1–0.2 mg/kg/h of cisatracurium) were infused during the operation.

All patients received standard monitoring with electrocardiography, and end-tidal carbon dioxide concentration, bispectral index, peripheral oxygen saturation, cerebral blood oxygenation, and invasive arterial monitoring of radial and femoral artery were applied. Central venous oxygen saturation monitoring (PreSep, Edwards Lifesciences, Irvine, CA, USA) was performed using an EV1000 monitoring platform (Edwards Lifesciences, Irvine, CA, USA) in order to monitor stroke volume, stroke volume index, cardiac output, cardiac index, central venous oxygen saturation, and systemic vascular resistance. Patients received transfusions as necessary/when required such that their hematocrit level was maintained at 25–30%. Norepinephrine (0.01–0.4 µg/kg/min) was used to maintain systolic blood pressure and mean arterial pressure above 90 and 60 mmHg, respectively. If norepinephrine was not effective, dobutamine, vasopressin, and epinephrine were considered.

LT and anesthesia were performed using conventional methods. Recipient hepatectomy was performed after cholecystectomy and total hepatectomy. A donor liver was inserted, and portal vein anastomosis was performed. After portal vein re-perfusion, hepatic artery anastomosis and re-perfusion were performed. Biliary reconstruction and bleeding control were then performed. Finally, the abdomen was closed.

Following surgery, the patient was transferred to the ICU. When their vital signs were stabilized and they exhibited no bleeding, extubation was performed if they could recover consciousness and spontaneous breathing.

### 2.5. Statistical Analysis

Data were analyzed using Statistical Analysis System version 9.3 (SAS Institute, Cary, NC, USA) and R software version 3.3.2 (R Project for Statistical Computing, Vienna, Austria).

Data are expressed as mean ± standard deviation, median (25th to 75th percentile), or number of patients (%). The normality of the data was assessed using the Shapiro–Wilk test or Kolmogorov–-Smirnov test. The independent *t*-test or Wilcoxon rank-sum test was used to compare continuous variables between the two groups. The chi-square test or Fisher’s exact test was used to compare categorical variables between the two groups. A *p* value < 0.05 was considered statistically significant.

Receiver operating characteristic (ROC) curve analysis was performed in order to assess the significance of the relationship between preoperative bilirubin level and postoperative delirium after LT. The sensitivity and specificity were established in order to use the potential cutoff values and thereby distinguish the association of delirium with preoperative bilirubin level.

The association between preoperative bilirubin level and postoperative delirium was assessed using univariate and multivariate analyses. Typical factors associated with postoperative delirium after LT were analyzed by univariate analysis, and multivariate analysis was performed using seven factors with a *p* value < 0.05 in the univariate analysis and clinical significance.

## 3. Results

A total of 325 patients were ultimately included in this study (Figure 1), 256 and 69 of whom were categorized into the Control group and the Delirium group, respectively. In this study, 69 (21.23%) patients had postoperative delirium after LT.

Table 1 shows the baseline characteristics of patients undergoing LT. The Delirium group had lower BMI levels (*p* = 0.001), and a higher proportion of patients in the Delirium group required LT due to alcoholic cirrhosis (*p* < 0.001) than those in the Control group. Moreover, patients in the Delirium group had a higher MELD score than those in the Control group (*p* < 0.001) and a higher proportion of patients in the Delirium group experienced cardiovascular accidents before surgery (*p* = 0.045). In addition, a higher proportion of patients in the Delirium group took diuretics (*p* = 0.003), insulin (*p* = 0.038), or psychiatric drugs (*p* = 0.003). Furthermore, a higher proportion of patients in the Delirium group suffered from sleep disorders (*p* = 0.001), had experienced previous delirium (*p* = 0.022), or exhibited mobility limitations (*p* = 0.022).

Table 2 shows the preoperative laboratory data of patients undergoing LT. Patients in the Delirium group had lower preoperative hemoglobin levels (*p* < 0.0001), a lower preoperative platelet count (*p* = 0.017), lower preoperative sodium levels (*p* = 0.005), and higher creatinine levels (*p* < 0.0001) than those in the Control group. Moreover, patients in the Delirium group had higher preoperative bilirubin levels (*p* < 0.0001), and 54 (78.26%) patients in this group had preoperative bilirubin levels above 3.5 μmol/L (*p* < 0.0001).

Table 3 shows the perioperative data of patients undergoing LT. There was no difference in anesthetic duration between the two groups (*p* = 0.506). However, estimated blood loss was higher in patients in the Delirium group (*p* < 0.0001), and a higher proportion of patients in the Delirium group required packed red blood cell (*p* < 0.0001), fresh frozen plasma (*p* < 0.0001), or platelet (*p* = 0.008) transfusions.

Table 4 shows the postoperative data of patients undergoing LT. There was no difference in postoperative hemoglobin levels between the two groups (*p* = 0.837). However, patients in the Delirium group exhibited higher postoperative bilirubin and creatinine levels (*p* < 0.0001) and lower postoperative protein (*p* = 0.002) and albumin (*p* = 0.019). Moreover, patients in the Delirium group spent longer in the ICU (*p* < 0.0001) and exhibited a longer time from surgery to discharge (*p* < 0.0001) than those in the Control group.

**Figure 2:**
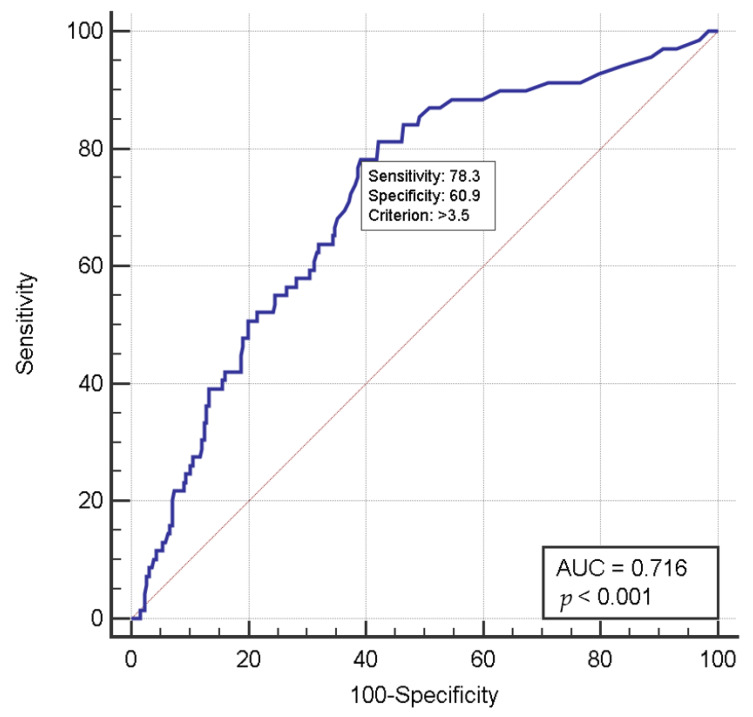
Diagnostic performance (receiver operating characteristic curve) of serum bilirubin level for predicting the incidence of postoperative delirium in liver transplant patients.

The cutoff value of preoperative total bilirubin was 3.50 mg/dL with a sensitivity of 78.3% and specificity of 60.9% (area under the curve, 0.716; 95% confidence interval, 0.783–0.882; *p* < 0.001; Figure 2).

In the univariate analysis, preoperative bilirubin, body mass index, alcoholic liver disease, diuretic use, insulin use, psychiatric medication use, previous delirium, preoperative hemoglobin, preoperative sodium, preoperative creatinine, estimated intraoperative blood loss, total volume of fluid administered intraoperatively, number of packed red blood cell units transfused, and postoperative albumin were associated with postoperative delirium after LT, as shown in Table 5. The multivariate analysis revealed that preoperative bilirubin, body mass index, diuretic use, and preoperative creatinine were all independent risk factors for postoperative delirium after LT.

Table 6 shows analysis of postoperative delirium using four kinds of models with clinical significance. Preoperative bilirubin levels above 3.5 mg/dL were significantly associated with postoperative delirium in all four models (*p* < 0.05).

## 4. Discussion

In this retrospective analysis of LT recipients, a preoperative serum bilirubin above 3.5 mg/dL was associated with postoperative delirium. Patients with postoperative delirium had higher preoperative bilirubin levels than those without, and 78% of patients with postoperative delirium had preoperative bilirubin levels above 3.5 mg/dL. In the multivariate analysis, preoperative hyperbilirubinemia was an independent risk factor for postoperative delirium (*p* = 0.002).

In patients undergoing LT, neurological events are relatively common due to the high incidence of underlying conditions in these individuals, specifically preoperative HE, metabolic impairments, a history of alcoholism, and severe ascites. Impaired liver function due to liver cirrhosis causes electrolyte imbalances, kidney injury, infection, and HE [2,13]. In addition, LT is a long and complicated surgical procedure and is associated with extensive intraoperative blood loss, frequent blood transfusions, long recovery periods in the ICU, and the postoperative use of immunosuppressive agents, all of which may contribute to the high incidence of the occurrence of postoperative neurological complications after LT [5,8]. Among neurological events, postoperative delirium is a common complication after LT, and the reported incidence of this condition varies from 9% to 32% [1,2,3]. Patients with delirium after LT require a prolonged stay in the ICU and prolonged mechanical ventilation and exhibit increased mortality. These patients are immobilized for longer, which slows the onset of postoperative exercise and makes it difficult for early rehabilitation treatment to be initiated [14]. Although the course of postoperative delirium is usually reversible, it has also been associated with long-term neurologic deterioration such as cognitive impairment and dementia [15,16]. As a result, delirium after LT is associated with increased post-transplant mortality and morbidity and high medical expenses. Recently, the number of patients undergoing LT and the survival rate of recipients have increased; therefore, interest in post-transplant management has also increased. As such, it is essential that patients at high risk of developing postoperative delirium are identified.

Previously reported predictive factors for postoperative delirium include preoperative ascites, HE, sodium or magnesium imbalance, and MELD score [3,9]. MELD score has often been used as an indicator to demonstrate the severity of liver function impairment, prioritize liver allocation, and predict the mortality risk of patients scheduled for LT [17]. MELD score is calculated using a patient’s serum bilirubin, serum creatinine, and prothrombin time, but it is not clear which of these components are associated with the postoperative delirium.

Bilirubin is a lipid-soluble substance produced as the final product of heme metabolism in mammals. It easily crosses cell membranes, including the blood–brain barrier (BBB), and is reported to have both neuroprotective and neurotoxic effects [10,18]. Most bilirubin binds to albumin, but unbound bilirubin passes through the BBB, attaches to nerve cells, and exerts cytotoxic effects. Neurological symptoms caused by hyperbilirubinemia are common in newborns and diminish after maturation of the BBB [11]. However, patients with liver cirrhosis, decreased albumin production, or drug-induced bilirubin displacement from albumin binding sites are susceptible to/vulnerable to neurologic complications [19]. In a previous study, administration of drugs that interfere with *p*-glycoprotein function, namely rifampin, ceftriaxone, verapamil, and propranolol, promoted bilirubin accumulation in the brain of rats. Thus, there is concern about the possible effects of these drugs in humans who have or are at high risk of hyperbilirubinemia. Some of the major intracellular mechanisms by which bilirubin exerts its neurotoxic effects include cell membrane perturbation, DNA damage, modification of synaptic transmission, increased cytokine release, inhibition of neurotransmitter signaling, and apoptosis [10,20].

The prevention and early detection of delirium is important for the treatment and management of patients with LT to decrease post-LT morbidity and mortality. One of the characteristic aspects of postoperative delirium is that there is a time delay between the operation and the onset of delirium. There are no apparent mental symptoms during this period, and delirium typically develops within the first 24–48 h after LT. On average, delirium resolves within 5 days of surgery [3]. There are a number of methods available for diagnosing postoperative delirium, among which the DSM-IV and International Classification of Diseases, 10th Revision criteria are the most widely used [21]. In this study, the DSM-IV criteria were used to diagnose delirium. The quick resolution of delirium is likely due to liver function normalizing postoperatively and the transplanted liver beginning to clear metabolites [9].

There are some limitations of the present study. First, some variables that can potentially cause delirium after LT may not have been considered/assessed/analyzed due to the retrospective nature of this study. Second, this was a single-center study that included a small number of patients, so our findings are not definitive evidence of an association between preoperative hyperbilirubinemia and postoperative delirium. Third, delirium after LT is a clinical diagnosis, there are relevant issues about it. Indeed, LT per se can trigger delirium in any previously subclinical psychiatric disease. Moreover, because liver failure is often followed by HE, it is very difficult or impossible to distinguish cases with preexisting psychiatric disease from those with real HE. Therefore, although efforts were made to exclude patients who were diagnosed with HE or who were unable to have their mental status evaluated, it is difficult to completely rule out the effects of preclinical psychiatric disease or HE.

## 5. Conclusions

In conclusion, we found that preoperative hyperbilirubinemia appears to be an independent risk factor for delirium after LT. However, high bilirubin is one of the main signs of advanced liver failure. Therefore, the postoperative delirium may possibly be due to the general severity of the disease and may not be attributable to a single sign, such as bilirubin value. Therefore, prospective randomized control studies are required to confirm this association between hyperbilirubinemia and postoperative delirium in LT recipients.

## Figures and Tables

**Table 1:** Patient characteristics in patients undergoing liver transplantation.

	Control Group (*n* = 256)	Delirium Group (*n* = 69)	*p* Value
Age (years)	53.20 ± 8.20	54.12 ± 7.65	0.404
Sex (F/M)	187 (73.05)/69 (26.95)	48 (69.57)/21 (30.43)	0.566
Body mass index (kg/m^2^)	22.47 ± 3.14	20.93 ± 3.43	0.001 *
Cause of LT			<0.0001 *
Alcoholic cirrhosis	56 (21.88)	35 (50.73)	
HBV and HCV	168 (65.63)	24 (34.78)	
NBNC LC	10 (3.91)	5 (7.25)	
Toxic hepatitis	16 (6.25)	3 (4.35)	
Primary biliary cirrhosis	6 (2.34)	2 (2.90)	
MELD score	18.35 ± 11.50	28.32 ± 11.71	<0.0001 *
Hypertension	51 (19.92)	12 (17.39)	0.637
Diabetes mellitus	70 (27.34)	14 (20.29)	0.235
Ischemic heart disease	2 (0.78)	3 (4.35)	0.066
Congestive heart failure	1 (0.39)	1 (1.45)	0.380
Cerebrovascular accidents	0 (0.00)	2 (2.90)	0.045 *
Cardiac arrhythmia	6 (2.34)	3 (4.35)	0.407
With diuretics	47 (18.36)	24 (34.78)	0.003 *
With insulin	11 (4.30)	8 (11.59)	0.038 *
With psychiatric medication	10 (3.91)	10 (14.49)	0.003 *
Sleep disorder	22 (8.59)	16 (23.19)	0.001 *
Previous delirium	8 (3.13)	7 (10.14)	0.022 *
Physical activity(self/assistance)	219 (85.55)/37 (14.45)	51 (73.91)/18 (26.09)	0.022 *

Values are presented as mean ± SD or number (%). C group: those without delirium after LT, D group: those with delirium after LT. LT: liver transplantation, HBV: hepatitis B virus, HCV: hepatitis C virus, NBNC LC: non-HBV non-HCV liver cirrhosis, MELD: model for end-stage liver disease. * *p* < 0.05 compared between groups.

**Table 2:** Preoperative laboratory data in patients undergoing liver transplantation.

	Control Group (*n* = 256)	Delirium Group (*n* = 69)	*p* Value
Hemoglobin (g/dL)	10.98 ± 2.30	9.62 ± 1.95	<0.0001 *
Platelet (×10^3^/mL)	68.50 (46.00, 101.25)	52.00 (37.00, 79.00)	0.017 *
Creatinine (mg/dL)	0.79 (0.65, 0.99)	1.14 (0.72, 1.92)	<0.0001 *
AST (unit/L)	47.00 (34.00, 75.25)	63.00 (40.00, 117.00)	0.001 *
ALT (unit/L)	31.00 (20.00, 49.00)	34.00 (21.00, 52.00)	0.446
Protein (g/dL)	6.04 ± 0.85	5.69 ± 0.96	0.003 *
Albumin (g/dL)	3.16 ± 0.57	3.11 ± 0.57	0.527
Bilirubin (mg/dL)	2.40 (1.30, 12.62)	16.60 (3.90, 29.00)	<0.0001 *
≤3.5 mg/dL	156 (60.94)	15 (21.74)	<0.0001 *
>3.5 mg/dL	100 (39.06)	54 (78.26)	
PT (INR)	1.48 (1.22, 2.12)	1.98 (1.65, 2.80)	<0.0001 *
Use insulin during operation (unit)	5.00 (0.00, 10.00)	5.00 (0.00, 10.00)	0.851
Sodium (mEq/L)	136.66 ± 5.32	134.58 ± 5.66	0.005 *
Potassium (mEq/L)	3.97 ± 0.51	4.03 ± 0.74	0.537

Values are expressed as mean ± SD, number (percent) or median (interquartile range). C group: those without delirium after LT, D group: those with delirium after LT. AST: aspartate aminotransferase, ALT: alanine aminotransferase, PT (INR): prothrombin time (international normalized ratio). * *p* < 0.05 compared between groups.

**Table 3:** Perioperative factors in patients undergoing liver transplantation.

	Control Group (*n* = 256)	Delirium Group (*n* = 69)	*p* Value
Anesthetic duration (h)	10.88 ± 2.30	10.65 ± 3.21	0.506
Estimated blood loss (L)	2.50 (1.50, 4.55)	4.0 (2.50, 6.00)	<0.0001 *
Total fluid intake (L)	7.04 (5.39, 9.60)	9.22 (7.33, 12.55)	<0.0001 *
pRBC (unit)	5.15 ± 5.43	9.30 ± 6.13	<0.0001 *
Fresh frozen plasma (unit)	5.08 ± 5.62	8.88 ± 6.02	<0.0001 *
Cryoprecipitate (unit)	0.00 (0.00, 0.00)	0.00 (0.00, 0.00)	0.525
Platelet (unit)	0.00 (0.00, 0.00)	0.00 (0.00, 2.00)	0.008 *

Values are expressed as mean ± SD or median (interquartile range). C group: those without delirium after LT, D group: those with delirium after LT. pRBC: packed red blood cells. * *p* < 0.05 compared between groups.

**Table 4:** Postoperative data in patients undergoing liver transplantation.

	Control Group (*n* = 256)	Delirium Group (*n* = 69)	*p* Value
Hemoglobin (g/dL)	9.04 ± 1.48	9.08 ± 1.55	0.837
Platelet (×10^3^/mL)	52.50 (39.00, 76.25)	48.00 (36.00, 67.00)	0.232
PT (INR)	1.75 (1.52, 1.98)	1.78 (1.55, 2.04)	0.293
Bilirubin (mg/dL)	4.10 (2.10, 7.03)	6.40 (4.00, 10.50)	<0.0001 *
Protein (g/dL)	5.29 ± 0.67	5.01 ± 0.69	0.002 *
Albumin (g/dL)	3.66 ± 0.47	3.51 ± 0.52	0.019 *
Creatinine (mg/dL)	1.05 ± 0.49	1.30 ± 0.54	<0.0001 *
Sodium (mEq/L)	139.54 ± 3.72	138.68 ± 3.85	0.093
Potassium (mEq/L)	4.00 ± 0.40	4.07 ± 0.45	0.206
Stay in intensive care unit (days)	7.00 (5.00, 11.00)	12.00 (7.00, 18.00)	<0.0001 *
Infection	11 (4.30)	7 (10.14)	0.074
Time from surgery to discharge (days)	27.00 (22.00, 38.00)	40.00 (28.00, 54.00)	<0.0001 *

Values are expressed as mean ±SD, number (%) or median (range). C group: those without delirium after LT, D group: those with delirium after LT. PT (INR): prothrombin time (international normalized ratio). * *p* < 0.05 compared between groups.

**Table 5:** Logistic regression analysis.

Predictors	OR	95% CI		*p* Value *	OR	95% CI		*p* Value ^†^
		Lower	Upper			Lower	Upper	
Bilirubin (3.5<)	5.616	3.007	10.489	<0.0001 *	2.864	1.381	5.941	0.005 ^†^
Age	1.015	0.981	1.050	0.403				
Sex	0.843	0.471	1.510	0.567				
Body mass index	0.847	0.770	0.932	0.001 *	0.853	0.770	0.944	0.005 ^†^
Alcoholic liver disease	3.676	2.106	6.419	<0.0001 *	1.630	0.850	3.127	0.141
with Diuretics	2.372	1.318	4.269	0.004 *	2.181	1.105	4.303	0.025 ^†^
with Insulin	2.921	1.126	7.575	0.028 *				
Psychiatric medication	4.169	1.659	10.477	0.002 *	2.813	0.942	8.400	0.064
Previous delirium	2.089	1.101	3.963	0.024 *				
Hb before surgery	0.736	0.639	0.849	<0.0001 *				
Albumin before surgery	0.859	0.538	1.373	0.526				
Na before surgery	0.932	0.887	0.979	0.005 *				
Cr before surgery	1.903	1.427	2.538	0.0001 *	1.504	1.097	2.063	0.011 ^†^
Anesthetic duration	0.963	0.863	1.076	0.505				
Intraoperative EBL (L)	1.165	1.050	1.292	0.004 *				
Total_fluid (L)	1.089	1.033	1.148	0.002 *				
pRBC (pint)	1.123	1.071	1.177	<0.0001 *	1.043	0.985	1.104	0.146
Hb after surgery	1.019	0.853	1.217	0.836				
Albumin after surgery	0.523	0.303	0.905	0.021 *				
Na after surgery	0.941	0.876	1.010	0.094				
K after surgery	1.512	0.796	2.873	0.207				
Infection	2.515	0.936	6.752	0.067				

OR: odds ratios, CI: confidence interval, BMI: body mass index, Hb: hemoglobin, Na: sodium, Cr: creatinine, EBL: estimated blood loss, pRBC: packed red blood cells, K: potassium. * *p* value of univariate crude data. ^†^ *p* value of multivariate crude data.

**Table 6:** Variable analysis of postoperative delirium in patients undergoing liver transplantation.

		Odds Ratio	95% Confidence Interval		*p* Value
			Lower	Upper	
Univariate	Bilirubin (3.5<)	5.616	3.007	10.489	<0.0001 *
Multivariable analysis					
Model 1	Bilirubin (3.5<)	2.864	1.381	5.941	0.005 ^†^
Model 2	Bilirubin (3.5<)	2.794	1.340	5.828	0.006 ^†^
Model 3	Bilirubin (3.5<)	3.266	1.617	6.597	0.001 ^†^
Model 4	Bilirubin (3.5<)	3.195	1.574	6.484	0.001 ^†^

Models were adjusted for bilirubin, BMI, alcoholic liver disease, with diuretics, psychiatric medication, preoperative creatinine, intraoperative packed red blood cells, and postoperative infection. Model 1: except postoperative infection, Model 2: with all factors, Model 3: except alcoholic liver disease and postoperative infection, Model 4: except alcoholic liver disease. * *p* value of univariate crude data, ^†^ *p* value of multivariate crude data.
